# Study on Synergistic Anti-Inflammatory Effect of Typical Functional Components of Extracts of Ginkgo Biloba Leaves

**DOI:** 10.3390/molecules28031377

**Published:** 2023-02-01

**Authors:** Lihu Zhang, Xianying Fang, Jihu Sun, Erzheng Su, Fuliang Cao, Linguo Zhao

**Affiliations:** 1 Department of Pharmacy, Jiangsu Vocational College of Medicine, Yancheng 224005, China; 2 Jiangsu Co-Innovation Center of Efficient Processing and Utilization of Forest Resources, Nanjing Forestry University, Nanjing 210037, China; 3 Co-Innovation Center for Sustainable Forestry in Southern China, Nanjing Forestry University, Nanjing 210037, China; 4 College of Chemical Engineering, Nanjing Forestry University, Nanjing 210037, China

**Keywords:** ginkgo biloba extract (EGB), synergism, anti-inflammatory, ginkgo flavone, ginkgolide

## Abstract

There are some differences in the anti-inflammatory activities of four typical components in EGB (extracts of ginkgo biloba leaves), and there is also a synergistic relationship. The order of inhibiting the NO-release ability of single functional components is OA > GF > OPC > G. Ginkgolide (G), proanthocyanidins (OPC), and organic acids (OA) all have synergistic effects on ginkgo flavonoids (GF). GF:OA (1:9) is the lowest interaction index among all complexes, showing the strongest synergy. The anti-inflammatory mechanism of the compound affects the expression of p-JNK, p-P38, and p-ERK1/2 proteins by inhibiting the expression of iNOS and COX2 genes on NFKB and MAPK pathways. This also provides a research basis for the development of anti-inflammatory deep-processing products of EGB.

## 1. Introduction

Ginkgo biloba extract (EGB) contains many typical functional components such as ginkgo flavonoids (GF), ginkgolide (G), proanthocyanidins (OPC), and organic acids (OA) [1,2]. Its clinical application is based on EGB. The research on its function and the positioning of its efficacy is almost the entirety of EGB. Ginkgo biloba extract contains nearly 70% of other components in addition to total flavonoids and ginkgolide of EGB. Whether these "other components" have anti-inflammatory effects in ginkgo biloba extract and preparation and whether they have synergistic effects on the anti-inflammatory activities of Ginkgo flavonoids and ginkgolides have not been deeply studied.

Studies have shown that EGB has anti-inflammatory effects in many aspects. EGB activates heme oxygenase to prevent inflammation and prevent diabetic nephropathy. EGB can significantly reduce TNF in glomerulus-α and increase the accumulation of IL-6, fibronectin, and lipids. The high glucose level stimulated the podocyte to absorb the low-density lipoprotein, reduced the production of ROS, and prevented the occurrence of diabetic nephropathy by activating NRF-2/HO-1 to protect human podocytes. Ginkgo biloba extract has anti-inflammatory activity on primary microglia stimulated by LPS. EGB can reduce neuroinflammatory activation by targeting the Cox/PGE2 pathway. This effect contributes to the treatment of Alzheimer’s disease and vascular and mixed Alzheimer’s disease [3,4,5,6]. Ginkgo biloba flavone, ginkgo biloba flavone monomer [7,8,9,10,11,12,13], ginkgo biloba lactone [14], and ginkgo lactone monomer have reported many antioxidant and anti-inflammatory effects. The results showed that ginkgo flavone significantly inhibited the abnormal expression of the Akt and p38 pathway in A549 cells stimulated by human neutrophil elastase. Inflammatory cells and cytokines (IL-8) were also reduced in treated mice. Ginkgo biloba biflavones can inhibit leukocyte elastase activity [15]. Ginkgolide A [16], ginkgolide B [17,18,19,20,21], ginkgolide C [22], ginkgolide J [23,24], ginkgolide K [24,25,26], and bilobalide [19] have reported anti-inflammatory activities. The early prediction results of network pharmacology show that organic acids and procyanidins have synergistic effects on the anti-inflammatory activity of ginkgo flavonoids [27]. On this basis, this paper intends to study the anti-inflammatory activity and synergistic effect of four typical functional components in EGB at the cellular level, optimize the proportion of the best functional components of anti-inflammatory using the equal radiation method and explore the anti-inflammatory molecular mechanism of the best compound at the protein and gene level. The research results are expected to provide new ideas for the quality positioning of ginkgo biloba extract and the in-depth development of products.

## 2. Results and Analysis

**Figure 1:**
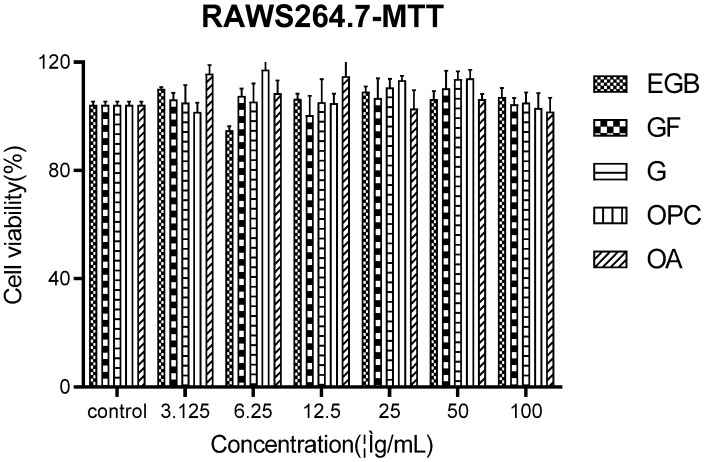
MTT assay for detecting the cytotoxicity of EGB to macrophages (all data were mean (±SD) of three independent experiments). G is the ginkgolide abbreviation, OPC is proanthocyanidins, OA is organic acids, and GF is ginkgo flavonoids.

The effects of different concentrations of EGB on Raws264.7 were detected by the MTT method and blank control. The results showed that the main effective components of EGB were against Raws274 in Figure 1. None of them had cytotoxicity.

### 2.1. The main Functional Components of EGB Inhibit LPS Stimulated Raws264 7 Cell Release of NO

**Figure 2:**
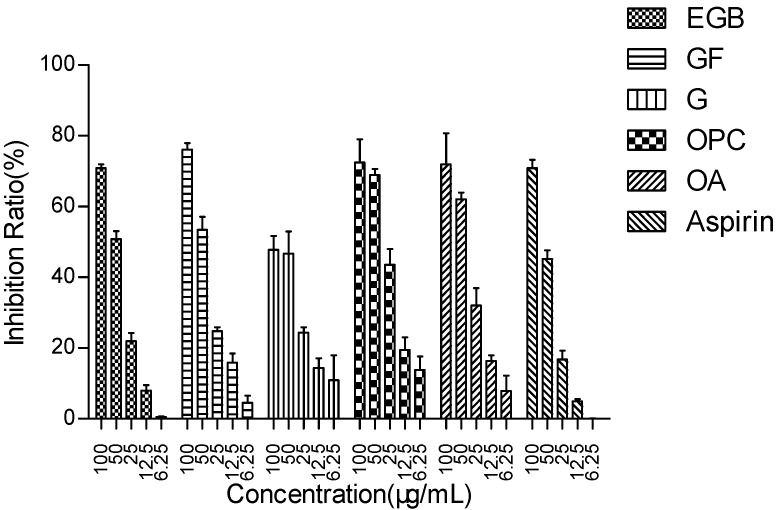
The inhibitory effect of EGB on LPS-induced NO in macrophages (with aspirin as a positive control). All data were averaged ± SD of three independent experiments.

To study the effect of EGB and other effective components on LPS stimulation of Raws264 7. Effect of NO release from cells. The results are shown in Figure 2 and Table 1.

The results showed that EGB had a concentration-dependent inhibitory effect on NO production, and the inhibitory rate was more than 70% at the concentration of 100 µg/mL, which was as good as Aspirin. The ability of the extract to inhibit the production of NO may be related to the inhibition of the activity or expression of nitric oxide synthase (iNOS). Ginkgo flavone glycoside and anthocyanin regulate iNOS by inhibiting nuclear transcription factor NFKB and modifying the signal pathway in immune cells [28,29,30].

### 2.2. The Main Efficacy Components of EGB on LPS Stimulated Raws264.7 Synergistic Effect of NO Production Inhibitory Activity

**Figure 3:**
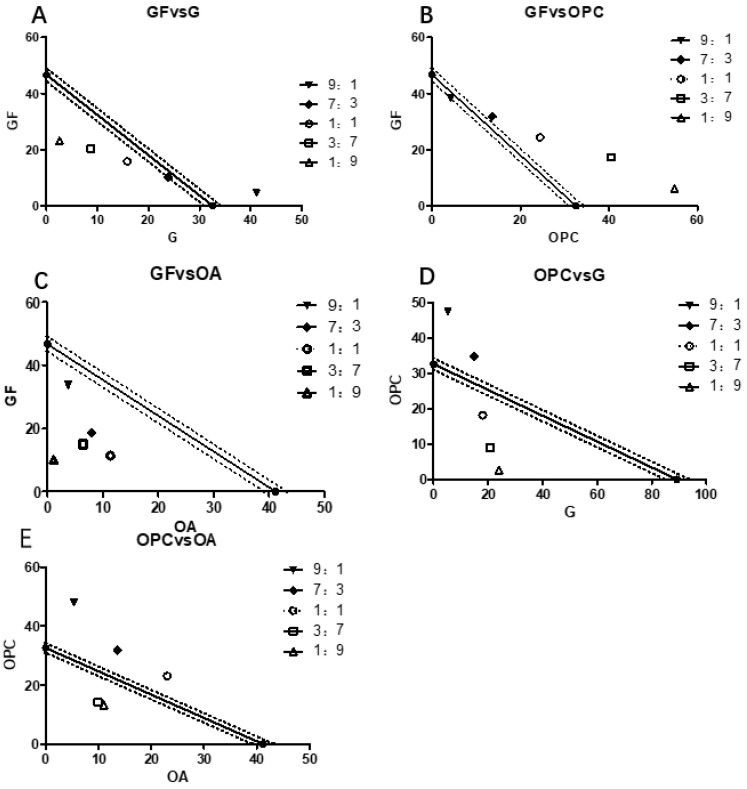
EGB synergistic effect of each functional component on LPS stimulation Raws264.7 and NO inhibitory activity. (Where, (**A**) is the isoradiometric figure of GF and G at different compounding proportions. (**B**) is the complex of GF and OPC in different proportions. (**C**) is the compound of GF and OA in different proportions. (**D**) is the complex of OPC and G in different proportions. (**E**) is the complex of OPC and OA in different proportions.).

The IC_50_ values were calculated by the compound ratios of ginkgo flavone (GF), ginkgolide (G), procyanidins (OPC), and organic acids (OA) of 9:1, 7:3, 1:1, 3:7, and 9:1, respectively, and then the isoradiation curve was made, as shown in Figure 3 and Table 2.

Through the statistical analysis of the data of the main functional components of EGB, it can be seen that the combination of G and GF or G and OPC has a synergistic effect on the Raws264.7 cells stimulated by LPS, and OPC had a significant synergistic effect on GF and OA. To further explore the molecular mechanism of its anti-inflammatory effect, the complex of GF:OA = 1:9 was selected to study the protein and gene levels of NFKB and MAPK pathways.

### 2.3. Comparison of the Effects of Complexes on the Expression of Related Proteins in NFKB and MAPK Pathways

**Figure 4:**
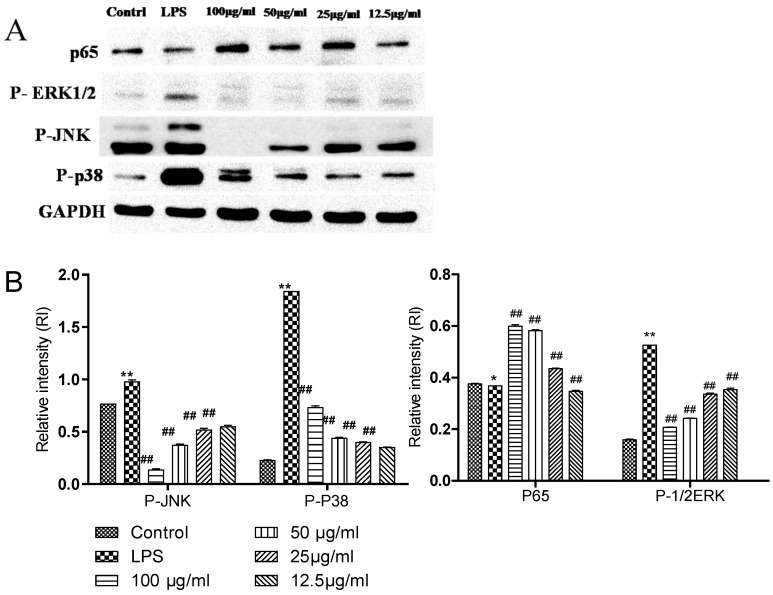
Effects of different concentrations of GF:OA = 1:9 on the proteins expression of P-p65 in NF-κB signaling pathway in LPS-induced RAW 264.7 cells. Effects of different concentrations of GF:OA = 1:9 on the proteins expression of P-JNK, P-p38α, and P-ERK1/2 in MAPK signaling pathway in LPS induced RAW 264.7 cells. Cells were pretreated with GF:OA = 1:9 (12.5, 25, 50, and 100 μg/mL) for 1 h, followed by LPS (1 μg/mL) stimulation for 24 h. The protein expression was determined by Western blot analysis, and GAPDH served as an internal control (**A**). Values represent the mean ± SD of the three independent experiments (* compared with the control, # compared with LPS, * *p* ≤ 0.05, and **/## *p* ≤ 0 01) (**B**).

The effect of complex (GF:OA = 1:9) on LPS-induced Raws264.7 cells that the effects of P65, p-JNK, p-p38, and p-ERK1/2 protein expression are shown in Figure 4. Compared with the blank control group, the expression of three proteins in the LPS group increased significantly, and compared with the LPS group containing compound 100 μg/mL, 50 μg/mL 25 μg/mL, and 12.5 μg/mL. The protein expression of p-JNK, p-p38, and p-ERK1/2 decreased significantly (*p* < 0.01), but the expression of the P65 protein did not change significantly.

### 2.4. Effect of the Complex on the Expression of Key Genes in the Anti-Inflammatory Pathway

**Figure 5:**
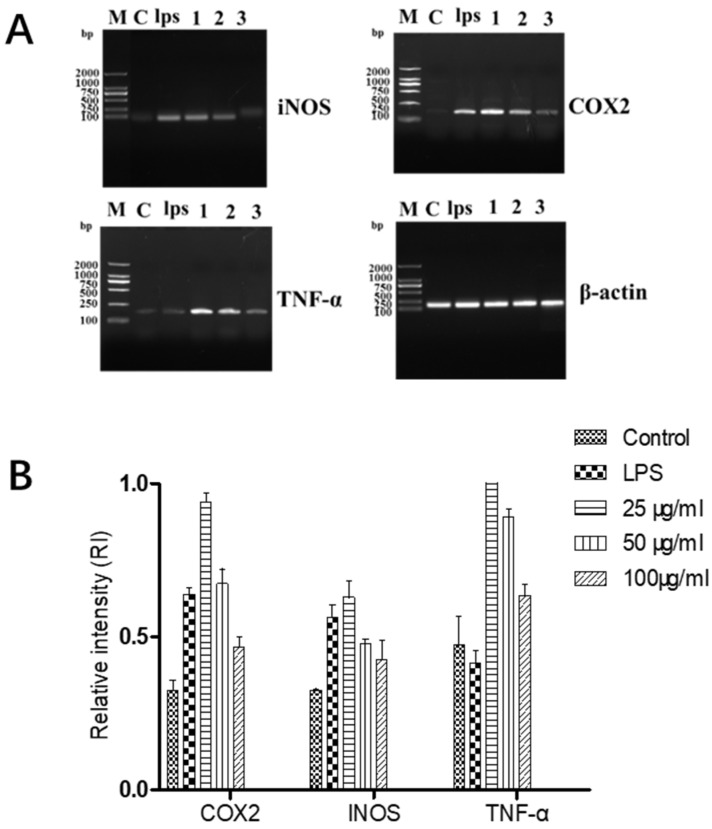
Effects of different concentrations of GF:OA = 1:9 on the genes’ expression of COX2, INOS and TNF in NF-κB signaling pathway in LPS-induced RAW 264.7 cells. Cells were pretreated with TFE (50, 100, and 200 μg/mL) for 1 h, followed by LPS (1 μg/mL) stimulation for 18 h (**A**). Values represent the mean ± SD of the three independent experiments (**B**).

The mixture of different concentrations (GF:OA = 1:9) acted on LPS-stimulated Raws264.7 cells. We extracted the total RNA and determined it by RT-PCR, and analyzed its grayscale with Image J software. The results are shown in Figure 5.

**Figure 6:**
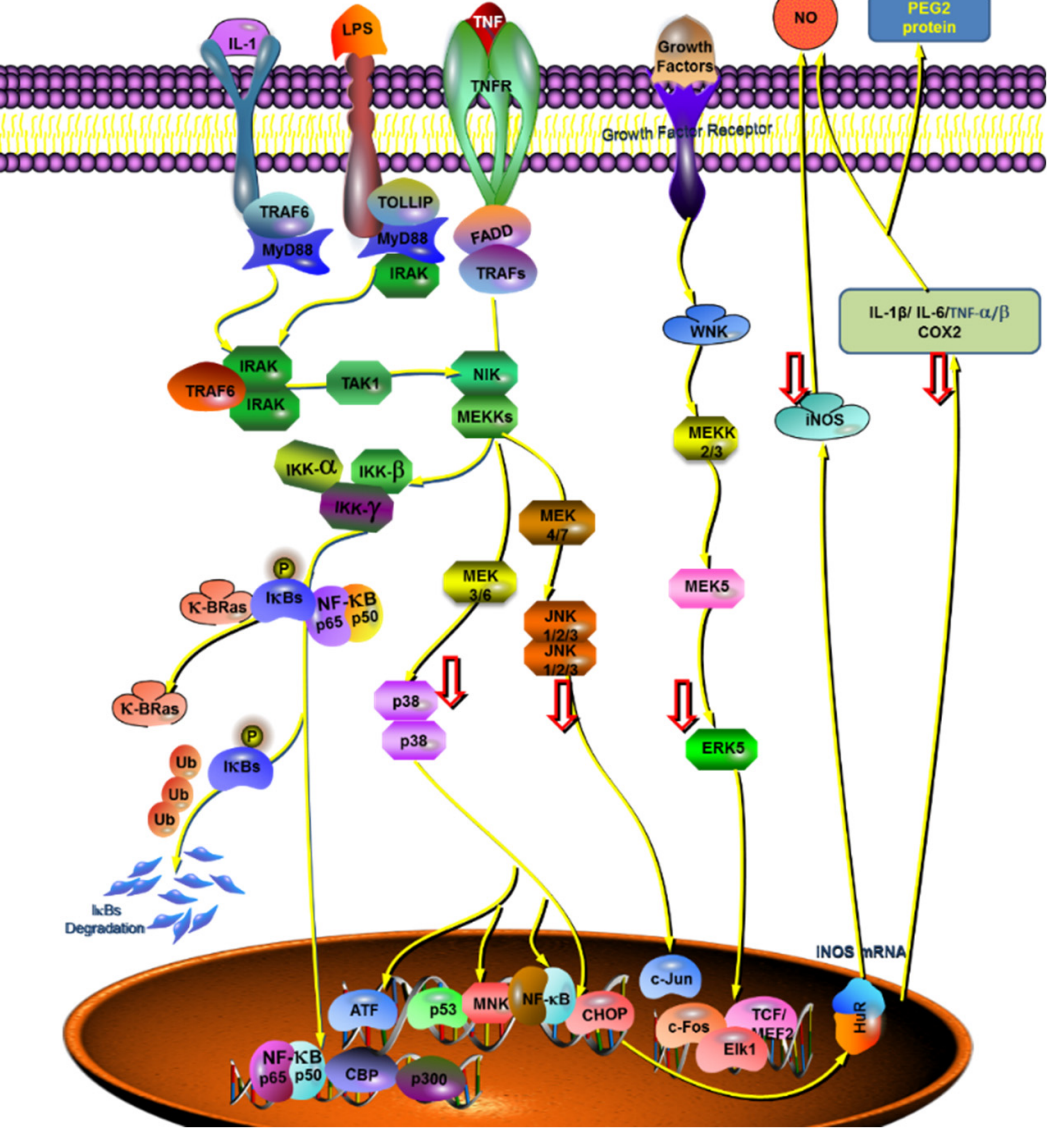
The proposed role of GF:OA = 1:9 on LPS-induced production of proinflammatory mediators and cytokines in RAW 264.7 cells. Cytokines, anti-inflammatory mediators, and key genes were mapped to the validated NFKB and MAPK pathways.

It can be seen from Figure 6 that the complex (GF:OA = 1:9) effects on LPS-induced Raws264.7 cells of INOS, COX2, and TNF-α. Compared with the LPS control group, the gene expression of iNOS and COX2 decreased significantly. The high-concentration compound did not affect TNF-α gene expression and did not decrease.

Based on the results of the whole anti-inflammatory experiment, the compound (GF:OA = 1:9) inhibited LPS stimulated Raws264.7 cells, and compared with the LPS group, the expression of p-JNK, p-P38, and p-ERK1/2 proteins decreased significantly, and the expression of COX2 and iNOS gene decreased significantly in high concentration complex, to play its anti-inflammatory role. Mapping of the cytokines, proteins, and genes that affect the complex’s anti-inflammatory responses to the established MAKP and NFKB pathways is shown in Figure 6.

Extracellular signal-regulated kinase ERK1/2 is a subfamily of the MAPK family. It is an important signal transduction molecule. It can be activated into phosphorylated ERK1/2 by a variety of stimulating factors such as cytokines, viruses, and G-protein-coupled receptor ligands to play its biological regulatory role [31]. Its upstream activators are two MAPK kinases, MEK1 and MEK2. MEK is a rare dual-specific kinase. When cells are stimulated by LPS, it can phosphorylate the two regulatory sites of Tyr and Thr and activate ERK1/2; MEK5 can be phosphorylated and activated, and then MKK3 and MKK6 can be phosphorylated and activated. Phosphorylated ERK1/2 can activate the gene expression activity of a variety of transcription factors. These activated transcription factors can be combined with cytokine gene promoters to up-regulate cytokines nitric oxide (NO) and TNF-α, gene expression of IL-1, IL-6, and matrix metalloproteinase. In this experiment, the relative expression of phosphorylated ERK1/2 was used to reflect the activation degree of ERK1/2. The results showed that the complex (GF:OA = 1:9) could inhibit the activation of ERK1/2 and down-regulate the expression of phosphorylated ERK1/2 protein by inhibiting the phosphorylation and activation of ERK1/2. The down-regulation of phosphorylated ERK1/2 protein expression could reduce the expression activity of many transcription factor genes to inhibit the expression of downstream cytokine genes and reduce the expression of NO and TNF-α. The production of inflammatory cytokines such as IL-1 decreased.

Common iNOS inducible NO synthase (iNOS) is mainly distributed in macrophages, epithelial cells, neutrophils, and other cells. Under normal conditions, iNOS does not or rarely exists in these cells. It is induced only when these cells are stimulated by substances such as LPS and inflammatory cytokines (IL-1, TNF, etc.) [32,33]. After iNOS is induced, its activity does not depend on calcium ion (Ca^2+^) and calmodulin (CAM) and can last from hours to days, working to catalyze the synthesis of a large amount of NO and finally mediate the occurrence and development of diseases such as inflammation. Studies have found that a variety of transcription factors and tyrosine kinases are involved in the regulation of iNOS expression [34]. An NFKB signaling pathway can regulate one of the two main pathways of iNOS gene expression [35]. The activation of the NFKB signaling pathway can induce the expression of iNOS and increase the expression of iNOS protein and the release of NO.

## 3. Experimental Method

### 3.1. Experimental Reagent and Instruments

#### 3.1.1. Experimental Reagents

Methanol, ethanol, Nanjing Jianghua chemical Glass Instrument Co., Ltd., Nanjing, China. DPPH (2,2-di (4-tert-octylphenyl)-1-picrylhydrazyl, PI, Sigma company, St. Louis, MO, USA. Phosphate buffer (PBS), RPMI-1640 medium, hyclone. Dimethyl sulfoxide, MTT, Nanjing Shengxing biological Co., Ltd., Nanjing, China. NO detection kit, annexin V-FITC cell apoptosis detection kit, WB and IP cell lysate, reactive oxygen species detection kit (ROS), BCA™ protein quantitative Kit (Pierce), trypsin digestive solution, Shanghai Biyuntian Biotechnology Co., Ltd., Shanghai, China. N,N′-Methylenebisacrylamide (BIS), sodium dodecyl sulfate (SDS), tris (hydroxymethyl) aminomethane (Tris), glycine, Amresco, Radnor, PA, USA. Ammonium persulfate, Nanjing Shengxing Biotechnology Co., Ltd., Nanjing, China. N,N,N′,N′-Tetramethylylenediamine (TEMED), Zhushi society of Heguang pure pharmaceutical industry, Osaka, Japan. Protein pre-staining, ladder, Thermo Scientific, Waltham, MA, USA. PVDF membrane, millipore, Burlington, MA, USA. HRP coupled anti-mouse and anti-rabbit IgG secondary antibodies and substrates (KPL, Manchester, UK). 20×LumiGLO^®^ Reagent and 20 ×Peroxid, GAPDH antibody, anti cPARP antibody, anti iNOS antibody, anti-ERK1/2-antibody, anti NFKB antibody, anti JNK antibody, anti-P38-antibody, anti AKT antibody, and other antibodies, cell signaling company, Danvers, MA, USA.

#### 3.1.2. Cell Lines

Mouse peritoneal macrophage Raws264.7 was purchased from the cell bank of the typical culture preservation Committee of the Chinese Academy of Sciences. DMEM (containing 10% FBS) was cultured under sterile conditions of 37 °C and 5% CO_2_. DMEM (containing 10% FBS) was cultured under sterile conditions of 37 °C and 5% CO_2_.

#### 3.1.3. Experimental Instruments

Digital display constant temperature water bath pot HH-4, Guohua Electric Appliance Co., Ltd., Changzhou, China. RV05 basic rotary evaporator, IKA group, Staufen, Germany. KH3200B ultrasonic cleaner, Kunhe Ultrasonic Instrument Co., Ltd., Shanghai, China. SBH-Ⅲ s circulating water multipurpose vacuum pump, Zhengzhou Changchengke industry and Trade Co., Ltd., Zhengzhou, China. WH861 vortex mixer, Taicang science and education equipment Co., Ltd., Taicang, China. BS124S electronic balance, Beijing saidoris Instrument System Co., Ltd., Beijing, China. HPLC Agilent 1260, Agilent, Santa Clara, CA, USA. Spectramax 190 microplate reader, Meigu molecular Instrument Co., Ltd., Shanghai, China. 3111 CO_2_ incubator, Thermo Fisher, Waltham, MA, USA. ClassⅡ type2 biosafety cabinet, Singapore Yisi High Tech Co., Ltd., Singapore. 1X71 fluorescence inverted microscope, Olympus, Tokyo, Japan. WV-GP240 ordinary optical microscope, Panasonic electron, Osaka, Japan. Allegra x-22r desktop high-speed centrifuge, Beckman Kurt Co., Ltd., Suzhou, China. QB-9006 constant temperature microplate fast oscillator, Shanghai Shupei Experimental Equipment Co., Ltd., Shanghai, China. Vertical plate electrophoresis apparatus, bio rad, Hercules, CA, USA. Rotating film instrument, bio rad, Hercules, CA, USA. TY-80R decolorization shaker, Nanjing Kaiji Biotechnology Co., Ltd., Nanjing, China. UVP gel imaging system, Beckman Kurt, Brea, CA, USA. UPLCLTQ-Orbitrap XL, Thermo, Waltham, MA, USA.

### 3.2. Preparation and HPLC-MS/MS Analysis of Typical Functional Components of EGB

According to the AB-8 resin method before the research group, obtain the ethanol eluent and concentrate it in a solid form [8]. Take 20 g of the dried mixture of ginkgo flavone and lactone, dilute it to 200 mL volume with dilute ethanol, stir and cool it to 12 °C. After standing, centrifuging, and filtering the clear liquid, extract the filtrate with 9:1 ethyl acetate n-hexane three times, and the amount of extractant each time will be one-third of the volume of the filtrate. Wash the organic phase with water twice; the amount of water used each time will be one-fifth of the volume of the organic phase. Add activated carbon with four times the mass of solid matter, stir, and adsorb for 1 h, then filter, separate the activated carbon, and wash with a small amount of ethyl acetate. The filtrate and washing solution is concentrated under reduced pressure to obtain ginkgolide samples. During the extracted aqueous phase, dissolve a small amount of the organic phase under reduced pressure, extract with 1/3 of the volume of water phase N-butanol three times, combine with the N-butanol phase, and wash with water. Distil under reduced pressure to separate n-butanol, then add ethanol and water, concentrate under reduced pressure and completely remove the solvent [36]. The concentrate is a ginkgo flavone sample. Procyanidins and organic acids are prepared by referring to the deep eutectic solvent (DESs) [37,38,39]. Weigh each component to prepare a concentration of 1 mg/mL and then dilute to 20–50 μg/mL. Four functional components are sampled at the same time to prepare HPLC or HPLC-ELSD test samples.

### 3.3. Isoradiometric Analysis

**Figure 7:**
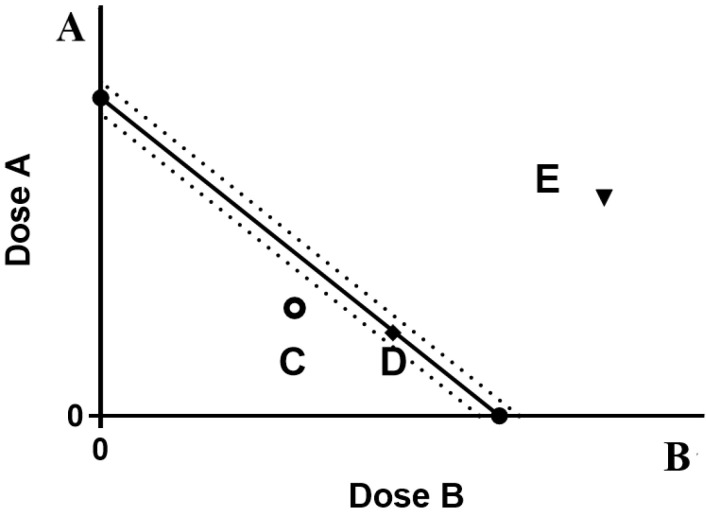
Isoradiation analysis of drugs A and B.

The typical components of the four kinds of EGB are configured as 10 mg/mL of mother liquor. According to the dosing concentration, the fixed ratio (volume ratio) of the two is 9:1, 7:3, 1:1, 3:7, and 1:9, 18 μL ginkgo flavone, and 2 μL ginkgolide plus rehydration are taken to 200 μL volume, the prepared concentration is 100 μg/mL of GF and G (volume ratio 9:1). The NO release inhibition rates of the above-mixed samples with different proportions were measured, respectively. The cell inhibition rate was measured by MTT assay. The IC_50_ value is calculated by Graphpad 8.0 software. The IC_50_ value of drug A is used as the ordinate, and the IC_50_ value of drug B is used as the abscissa. The IC_50a_ of compound A is used as the ordinate, and the IC_50b_ of compound B is used as the abscissa in the IC_50_ value of compound ab. The coordinate position points are determined, and their three functions are judged. The interaction coefficient is often used to evaluate the degree of synergy or antagonism(γ), as shown in Figure 7.

A: Dose of drug A, when used alone. B: Drug B alone is the dose of drug B. The line segment connecting two points AB is the contour line. The dotted line is the 95% confidence line. C indicates synergy, E indicates antagonism, and D indicates additivity.


$$\gamma =\frac{IC_{50Amix}}{IC_{50A}}+\frac{IC_{50Bmix}}{IC_{50B}}$$

IC_50amix_ and IC_50bmix_ are the IC_50_ values of antioxidants A and B in the compound group, respectively. IC_50a_ and IC_50b_ are the IC_50_ values of a and B antioxidants acting alone, if γ = 1. Indicates that the interaction is additive, if γ < 1. Indicates that the interaction is synergistic, the smaller the value, the stronger the synergy, if γ > 1. Indicates that the interaction is antagonistic.

### 3.4. Cell passage Culture Method

Raws264.7 cells are cultured in RPMI1640 medium (containing 10% neonatal bovine serum). Quickly discard the culture medium in the Petri dish, add 5 mL PBS along the cell-free wall, mix and discard it, and add a small amount of trypsin digestive solution to infiltrate and shed the cells. Add 6 mL of DMEM to blow evenly and transfer it to the centrifuge tube. After centrifugation at 4 °C and 800 rpm for 5 min, discard the supernatant. After washing with DMEM once, add an appropriate amount of the culture solution into the Petri dish containing DMEM to create a culture. The passage times can be determined according to different cell growth conditions.

### 3.5. Determination of NO Release by Griess Method

Griess reagent is composed of liquid A and liquid B. It is prepared and stored separately. When it is used temporarily, it is evenly mixed with the same volume ratio. Solution A: 2% sulfonamide, 5% phosphoric acid. Liquid B: 0.2% naphthalene ethylenediamine, both of which are dissolved in double-distilled water. The following describes the procedure. Take the logarithmic growth period of Raws264.7 cells, count them to create a density of 2 × 10^5^/mL, inoculate them into 96 well culture plates, and add 100 mL per well. After 24 h of culture, treat the cells with different concentrations of compounds and 500 ng/mL LPS. In the experimental group, three multiple wells were set at each concentration, and the culture medium containing DMSO was used as the control. After 48 h of the compound, 100% of the cell supernatant will be absorbed into the enzyme label plate; then add 100 μL of the evenly mixed Griess reagent. Measure the absorbance at 540 nm after 10 min of color development.

### 3.6. Extraction of Total Cell Protein

The cultured cells were collected by centrifugation, resuspended with 1 mL pre-cooled PBS, and transferred to an Eppendorf tube. Cells (3000 rpm × 5 min or 6000 rpm × 5 min) were collected by centrifugation for 0.5 min at 4 °C. The following describes the procedure. Discard the supernatant, disperse the cell precipitation, add 10^8^ cells/mL with pre-cooled cell lysate (including 1 mm PMSF, added before use) in proportion, and blow repeatedly and quickly. Stand on ice for 20–30 min. Remove × Cytoskeleton proteins by centrifugation for 5 min × 12,000 rpm at 4 °C. Carefully suck the supernatant out into the new Eppendorf tube. After protein quantification, store it at −70 °C for standby.

### 3.7. BCA total Protein Quantitative Method

Preparation of reaction solution: Add 200 μL mixture of liquid a and liquid B (liquid A: liquid B = 50:1 volume ratio) according to each sample, add 24 μL ddH_2_O and 1 μL protein of the sample to be tested, and mix well.

Preparation of standard curve: Aseptically suck 10 μL 2 mg/mL BSA standard solution, add 40 μL ddH_2_O, and mix well with S_6_ (400 μg/mL). Suck 25 μL S_6_ solution into 25 μL ddH_2_O and mix well with S_5_ (200 μg/mL). S_4_ (100 μg/mL), S_3_ (50 μg/mL), S_2_ (25 μg/mL), and S_1_ (12.5 μg/mL) are obtained by multiple dilutions, and S_0_ is 25 μL ddH_2_O (without protein). Add 200 μL reaction solution to each standard point and sample point (25 μL system), vortex mix for 15 s, react at 37 °C for 30 min, then cool to room temperature and measure the absorbance at 562 nm wavelength. Draw the standard curve, calculate the protein concentration of the sample (μg/mL), and convert it into μg/μL.

### 3.8. Western Blotting

Take 150 μL cells of the lysate (10 mM HEPES, 2 mM EDTA, 0.1% CHAPS, 5 mM DTT, and 1 mM PMSF). Use an ice bath for 0.5 h, perform centrifugation at 12000 rpm, for 2 min at 4 °C, and take the supernatant. The protein concentration is then determined by a BCA^TM^ protein quantitative kit. Prepare 10% SDS-PAGE adhesive according to the requirements of 50 μg protein loading, and perform electrophoresis at a low temperature of 4 °C, with a constant current of 20 mA for 1 h. After electrophoresis, transfer the protein on the gel to the PVDF membrane using the wet transfer method. After the transfer, place the membrane in a blocking buffer (2% free fat milk, 10 mM Tris-Cl, 50 mM NaCl, 0.1% Tween 20, pH 7.4) and incubate at room temperature for 2 h. The area without protein on the PVDF membrane is then closed. Cut the membrane to the required size according to the position indicated by the protein pre-dye ladder and incubate with the required primary antibody on the 4 °C converter overnight. The next day, place the membrane laced in a washing buffer (10 mm Tris-Cl, 50 mM NaCl, 0.1% Tween 20, pH 7.4) and gently shake and wash three times. The first and second times are for 5 min, and the third time should be for 10 min to remove the nonspecific binding primary antibody. Then co-incubate with properly diluted secondary antibody for 2 h at room temperature. Then, dilute the substrate in a ratio of 1:20 and incubate with the membrane for 1 min (ECL), and then photograph and record.

### 3.9. Reverse PCR (RT-PCR)

Total RNA extraction: the cultured passage cells are cultured at 4 °C. Collect the cells by centrifugation for 3000 rpm × 0.5 min, discard the supernatant, and add 1 mL of precooled Trizol for purging. Then transfer the cell suspension into a 1.5 mL Eppendorf tube and place it at room temperature for 5 min. Add 200 μL chloroform into each tube, shake violently for 15 s, and place at 15~25 °C for 3~15 min. The RNA is distributed in the upper aqueous phase after centrifugation for 15 min at a temperature of 4 °C and a speed of 10,000~12,000 rpm. Carefully suck the supernatant into the clean tube and discard the middle and lower organic phases. Then add an equal volume of isopropanol, shake gently, and place at room temperature for 5~10 min. Perform centrifugation at 4 °C and 12,000 rpm for 10 min. Discard the supernatant, add 1 mL 75% ethanol to wash the RNA precipitation, and place it at room temperature for 10~15 min. Perform centrifugation for 5 min at a temperature of 4 °C and a rotational speed of 7500 rpm. Discard the supernatant and dry the ethanol in the fume hood; add an appropriate amount. Dissolve RNA precipitation in DEPC water (if necessary, accelerate dissolution in 55~60 °C water bath for 10~15 min). After dissolving a small amount of DEPC water, measure the RNA concentration and OD 260/280 ratio. Store it at −70 °C for standby. If not, it should be used as RT-PCR as soon as possible. Detailed primer design and RT-PCR manipulation are attached in Supplementary Materials (Table S1).

### 3.10. Statistical Analysis Method

Graphpad prism 8 software (GraphPad Software Inc., San Diego, CA, USA) is used for all data analysis, such as anti-inflammatory factor and grey analysis. The data are expressed as mean ± standard deviation. Two-way ANOVA is used between groups, and the difference is statistically significant (*p* < 0.05). The densitometry data from Western blots are analyzed using ImageJ software (National Institutes of Health, Bethesda, MD, USA) to measure the optical density of each band.

## 4. Conclusions

The four typical components in EGB have great differences in anti-inflammatory activity, and their synergistic relationship is consistent with the previous prediction results. GF has strong anti-inflammatory activity. The order of single functional components inhibiting NO release is OA > GF > OPC > G. The results showed that the combination of G with GF or OPC had a synergistic effect, and the combination of OA with GF or OPC also had a synergistic effect. It is found that GF:OA = 1:9 is the lowest interaction index among all complexes, showing the strongest synergy. Its anti-inflammatory mechanism is through inhibiting the expression of iNOS and COX2 genes on NFKB and MAPK pathways and the expression of p-JNK, p-P38, and p-ERK1/2 proteins.

## Figures and Tables

**Table 1:** The IC_50_ value of NO in Raws264.7 cells of LPS inhibited by functional components of EGB.

Components	EGB	GF	G	OPC	OA	Aspirin
IC_50_ (μg/mL)	53.15 ± 1.62	46.72 ± 1.50	89.19 ± 0.80	32.56 ± 1.18	41.17 ± 1.31	58.46 ± 1.81

**Table 2:** Each functional component of EGB was combined with statistical analysis results.

Class of Complex	Proportion	The IC_50_ Value of Inhibiting NO Release in Raws264.7 Cells (μg/mL)			
		Theoretical Values	Measured Values	γ	P (95%)
GF + G	9:1	81.76	45.68 ± 1.21	0.56	0.008–0.022
	7:3	70.08	34.09 ± 0.76	0.49	0.011–0.037
	1:1	61.32	31.71 ± 1.03	0.52	0.032–0.055
	3:7	54.51	29.13 ± 0.66	0.53	0.009–0.017
	1:9	49.06	25.82 ± 0.81	0.53	0.011–0.026
GF + OPC	9:1	45.00	42.59 ± 1.26	0.95	0.015–0.037
	7:3	41.33	45.50 ± 1.31	1.10	0.006–0.018
	1:1	38.38	48.84 ± 1.52	1.27	0.027–0.046
	3:7	35.82	57.80 ± 1.18	1.61	0.041–0.082
	1:9	33.58	60.95 ± 2.01	1.82	0.021–0.035
GF + OA	9:1	48.67	37.49 ± 0.91	0.81	0.008–0.022
	7:3	44.90	26.68 ± 1.09	0.59	0.010–0.024
	1:1	43.77	22.71 ± 0.39	0.52	0.013–0.031
	3:7	42.69	21.40 ± 0.72	0.48	0.008–0.016
	1:9	41.66	11.24 ± 0.41	0.24	0.007–0.025
OPC + G	9:1	34.77	52.62 ± 2.16	1.51	0.031–0.047
	7:3	40.22	49.57 ± 1.20	1.23	0.014–0.026
	1:1	47.70	36.10 ± 0.91	0.76	0.020–0.047
	3:7	58.61	29.84 ± 0.63	0.51	0.009–0.015
	1:9	75.98	26.66 ± 0.77	0.35	0.013–0.028
OPC + OA	9:1	40.11	53.18 ± 2.13	1.60	0.012–0.026
	7:3	38.14	45.32 ± 1.45	1.30	0.031–0.048
	1:1	36.36	46.07 ± 1.38	1.27	0.033–0.080
	3:7	34.74	24.16 ± 0.29	0.68	0.012–0.047
	1:9	33.26	24.21 ± 0.41	0.67	0.013–0.024

## Data Availability

The basic data has been reflected in the paper, part of the original data is provided to the journal, and some other data can be shared by contacting us.

## Supplementary Materials

The following supporting information can be downloaded at: https://www.mdpi.com/article/10.3390/molecules28031377/s1, Table S1: Design of a series of EGB anti-inflammatory primers for each functional component.

## Sample Availability

Samples of the compounds involved in this paper and the company signed a contract, involving confidentiality is not convenient to provide.
