# Supplementary material for: Study on Synergistic Anti-Inflammatory Effect of Typical Functional Components of Extracts of Ginkgo Biloba Leaves
Source: Molecules. 2023 Feb 1;28(3):1377. doi: 10.3390/molecules28031377 (PMC9920934; doi:10.3390/molecules28031377)
Supplement: Supplementary file 1 [file molecules-28-01377-s001.zip › molecules-1844592-supplementary.pdf]

Supplementary Materials: Table S1

Primer design and RT-PCR manipulation

Determination of concentration: dilute RNA samples with DEPC-treated water. Detect the purity of RNA using a UV spectrophotometer. First, correct the zero point with DEPC-treated water. Read OD260 and OD280 values and the ratio of OD260/280. The OD260/280 ratio of pure RNA is usually 1.8~2.0. If the ratio is lower than 1.8, it indicates that there is protein pollution; in this case, extract it again with phenol/chloroform. If the ratio is lower than 2.0, it indicates salt pollution.

Primer design: according to the corresponding sequences in NCBI, the primers are designed with Primer 5.0 according to the design principle, then verified with Oligo 6.0, and handed over to Nanjing Kingsley company for synthesis. The primers are shown in Table 1 below:

Table S1. Design of a series of EGB anti-inflammatory primers for each functional component.

Gen	Sequence (5 '- 3')	Temperature℃	Base pair
COX-2	AGCCAGGCAGCAAATCCTT GGGTGGGCTTCAGCAGTAAT	60	40
iNOS	GGTGAAGGGACTGAGCTGTTACGTTCTCCGTTCTCTTGCAG	60	41
ERK1/2	GCGGCTGAAGGAGTTGATCAGGTAGGAGCAGGACCAGA	60	39
β-Actin	GTGCTATGTTGCTCTAGACTTCGATGCCACAGGATTCCATACC	60	43
CXCL1	CTTGCCTTGACCCTGAAGCTC	60	21
CXCL2	CCCCCTGGTTCAGAAAATCA	60	20
IL-6	ACACATGTTCTCTGGGAAATCGT	60	23
IL17	CCTCAAAGCTCAGCGTGTCC	60	20
IL-1β	CTCATTGTGGCTGTGGAGAA	60	20
IL-10	GGAGCAGGTGAAGAGTGATT	60	20
IL-2	GGATGCTCACCTTCAAATTT	60	20
IL-1α	TTCCCTCAACCAAACTATAT	60	20
TNF-a	TCAGTTCCATGGCCCAGAC	60	19

Reverse transcription reaction: according to the operational requirements of the RT-PCR kit, add dNTP 1 to microtubules on ice. Add 1-5 μL volume of RNA sample, add RTase-free water, inhibitor 0.5 μL,5×Buffer 4 μL. Make up deionized water to 20 μL. 42 °C, 20min, 99 °C, 5min, 4 °C infinite cycle reverse transcription reaction, sub packaging, and -80 °C preservation of cDNA.

PCR amplification: take the above 1 μL volume of cDNA sample, successively add reagent, 20 μ L total reaction system: premium 10 μL, cDNA 1 μ L, Primer 1 μL (mixed upstream and downstream primers), ddH2O 8 μL.
